# Early childhood screen time as a predictor of emotional and behavioral problems in children at 4 years: a birth cohort study in China

**DOI:** 10.1186/s12199-020-00926-w

**Published:** 2021-01-07

**Authors:** Wenwen Liu, Xiaoyan Wu, Kun Huang, Shuangqin Yan, Liya Ma, Hui Cao, Hong Gan, Fangbiao Tao

**Affiliations:** 1 Department of Maternal, Child, and Adolescent Health, School of Public Health, Anhui Medical University, No 81 Meishan Road, Hefei, 230032 Anhui China; 2 MOE Key Laboratory of Population Health Across Life Cycle, No 81 Meishan Road, Hefei, 230032 Anhui China; 3 NHC Key Laboratory of Study on Abnormal Gametes and Reproductive Tract, No 81 Meishan Road, Hefei, 230032 Anhui China; 4 Anhui Provincial Key Laboratory of Population Health and Aristogenics, No 81 Meishan Road, Hefei, 230032 Anhui China; 5 Maanshan Maternal and Child Healthcare Center, Maanshan, 243000 Anhui China

**Keywords:** Screen time, Psychological, Emotional and behavioral problems, Infants, Children, Longitudinal study

## Abstract

**Background:**

Previous studies have suggested that screen time (ST) has a negative effect on children’s emotional and behavioral health, but there are few longitudinal studies that have been conducted with infants and toddlers. This study sought to examine the effect of ST in early childhood on emotional and behavioral problems in children aged 4 years, based on a birth cohort study in China.

**Methods:**

A total of 2492 children aged 4 years were enrolled in this study. The parents and guardians of each child completed a questionnaire that included items eliciting information on children’s birth information, socio-demographic information at baseline, and ST at each follow-up. Emotional and behavioral problems were assessed using the Strengths and Difficulties Questionnaire (SDQ) at 4 years of age. Multivariate logistic analysis was used to explore the effects of ST on emotional and behavioral problems.

**Results:**

The percentages of children with ST > 0 h/day at age 0.5 years, ST > 2 h/day at age 2.5 years, and ST > 2 h/day at age 4 years were 45.7, 55.5, and 34.5% respectively. The prevalence of emotional and behavioral problems was 10.8%. ST at 6 months was a risk factor for emotional symptoms and hyperactivity at the age of 4 years. ST at age 2.5 years was a risk factor for hyperactivity at the age of 4 years. However, ST at age 4 years was a risk factor for total difficulties, conduct problems, peer problems, hyperactivity, and prosocial behavior.

**Conclusions:**

Higher ST exposure at early childhood is associated with later emotional and behavioral problems. In particular, sustained high ST exposure is a risk factor for behavioral problems. These findings suggested the importance of controlling ST to prevent the occurrence of emotional and behavioral problems in the early years.

**Supplementary Information:**

The online version contains supplementary material available at 10.1186/s12199-020-00926-w.

## Background

Emotional and behavioral problems are common psychological behavior deviation in childhood [1]. Approximately 10 to 20% of children and adolescents worldwide have one or more mental health problems, accounting for a significant portion of the global burden of childhood diseases [2]. Mental health problems not only reduce the health-related quality of life of children, but also continue to affect them throughout adult life [3, 4]. Many factors influence the mental health of children and adolescents, such as nighttime sleep duration [5], screen time (ST) [6], and physical activity [7]. Specifically, ST has been identified to be an important risk factor for the healthy growth of children.

With the development of the social economy, the use of various electronic products is increasingly entering the daily lives of people, and children are inevitably being exposed to electronic screens at earlier ages. The ST of children is increasing worldwide [8, 9], despite recommendations of the American Academy of Pediatrics (AAP) that children under 2 years old should avoid digital media use and that the ST of children older than 2 years should be limited to 2 h /day [10]. Mounting evidence has documented that many children start using screen media devices in infancy and increase their such media throughout childhood [11]; most children are already exposed to electronic screens at 6 months of age [12]. International statistics from 40 countries in Europe and North America show that more than 60% of children watch television for more than 2 h/day [13]. A survey of children aged 3 to 6 in 35 kindergartens in China revealed that 57.3% of the children were exposed to electronic screens exposed for more than 2 h/day [5]. Overexposure to screens in early childhood has become a common phenomenon, and its subsequent impact on children’s health is widespread, enough to warrant our attention.

There is a growing body of evidence which demonstrated that higher levels of ST are associated with negative emotional and behavioral problems in children and adolescents [14–16]. It is widely known that children are in a critical period of growth and development, especially in the first few years of their lives, with considerable plasticity [17]. Given that most of the infants are now exposed to the screen early in life [12], exploring their impact on the later healthy development of children is warranted. To date, most studies have focused on the association between school-age children and adolescents’ ST and their mental health. A small but growing number of studies have found the associations between ST and emotional and behavioral problems in infants and young children, such as a birth cohort study in Singapore found that infant television viewing has a negative association with cognitive skills at 4.5 years of age [18].

Many studies on ST apply cross-sectional designs, and longitudinal research is limited. Longitudinal studies are needed to identify such associations from early childhood to later childhood. Thus, the main purpose of the present study was to evaluate the association between ST in infants and toddlers with emotional and behavioral problems in a birth cohort study. We hypothesized that a high ST level in early childhood is a risk factor for emotional and behavioral problems. Furthermore, our birth cohort study design enabled us to examine the associations between sustained ST exposure and emotional and behavioral problems.

## Methods

### Study design and sample

**Fig. 1:**
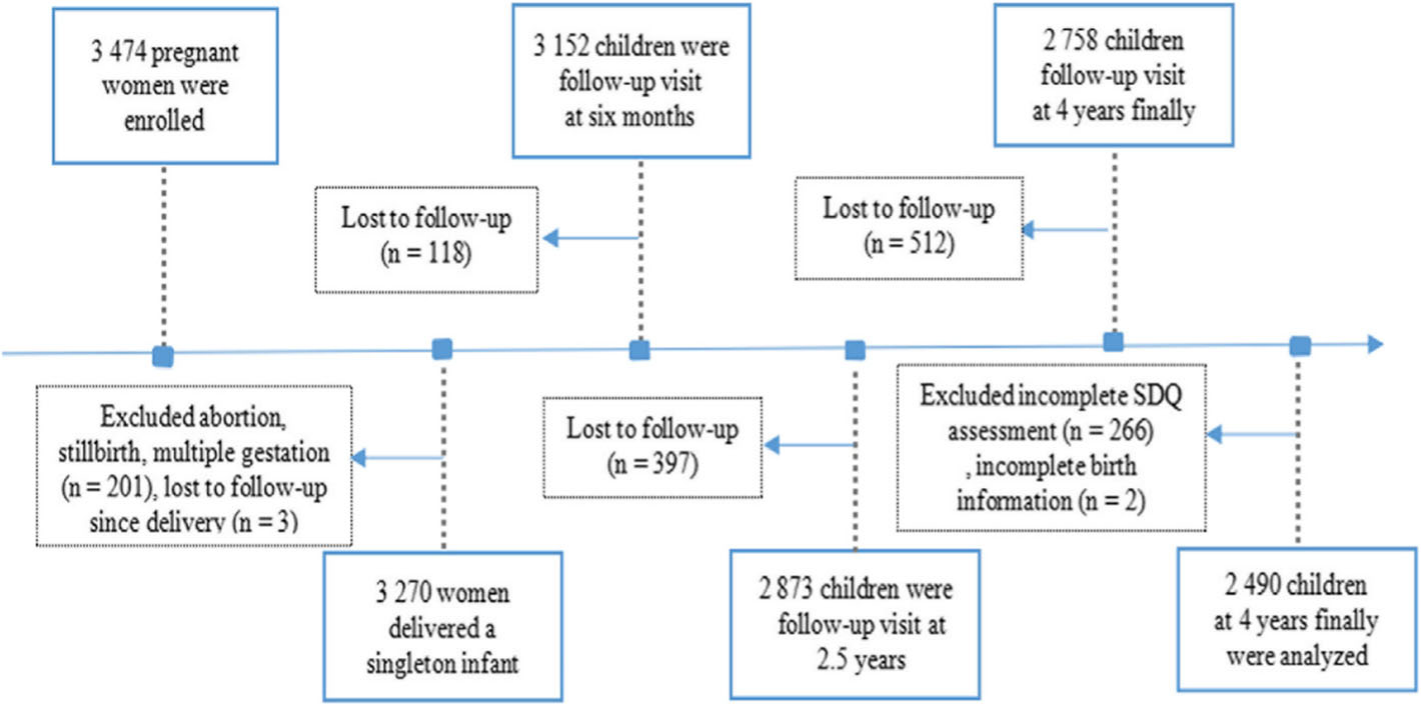
The follow-up procedures involved in this study (SDQ, Strength and Difficulties Questionnaire)

The children in this study were obtained from the Ma’anshan Birth Cohort (MABC) prospective cohort study, which was conducted in the city of Ma’anshan, Anhui province, China. Details on the procedures for the recruitment and exclusion of pregnant women have been described in previous research [19]. The MABC recruited 3474 pregnant women between May 2013 and September 2014. Children delivered by pregnant women enrolled in the cohort were automatically entered into the cohort for regular follow-ups. Questionnaires that included items from the Strengths and Difficulties Questionnaire (SDQ) as well as items on ST were distributed by maternal and child healthcare professionals when the children were examined at the ages of 0.5, 2.5, and 4 years respectively. At age 4 years of age, 2758 children remained in the survey. More than 30% of the blanks in the questionnaire were deemed invalid and eliminated, and incomplete SDQs and birth information were also directly excluded. The data of the final sample of 2490 children were obtained (Fig. 1), which included 1289 boys (51.8%) and 1201 girls (48.2%). Informed consent was obtained from the parents and guardians of the participants and the research protocol was approved by the ethics committee of the Anhui Medical University (Ethical approval number: 2008020).

### Exposure variables

Children’s ST was reported by their parents and guardians. The current study used two items to investigate the ST of infants aged 0.5 years: “How many hours does the child spends on average every day: (1) watching television and (2) using electronic products (mobile phones, tablets, computers, etc.)?”. Meanwhile, three items were used to investigate the ST of children aged 2.5 years: “How many hours does the child spends on average every day in the last month: (1) watching television? (2) using a cell phone? (3) using electronic products (computers or other electronic devices)?”. Total ST at the ages of 0.5 and 2.5 years were the sum of the values obtained for each item. The ST of children aged 4 years was obtained using the following items: “How many hours does the child spends: (1) watching television? (2) using computer? (3) using a cell phone? (4) using an iPad? (5) using other electronic devices?”. Specific hours and minutes were reported. These questions were repeated for weekdays and weekends. The total ST at age 4 years of age was the sum of the time spent on televisions, computer, cell phone, iPads, and other electronic device time during weekdays and weekends. The average daily ST was calculated using the formula (5/7 × [ST on weekdays] + 2/7 × [ST on weekends]). At the age of 0.5 years, an ST of 0 h/day was categorized as low and an ST of > 0 h/day high, while at the ages of 2.5 years and 4 years, an ST of ≤ 2 h/day was categorized as low and an ST of > 2 h/day high, according to the recommendation of the AA P[10].

Continuous high ST was defined as an ST > 0 h/day at age 0.5 years, ST > 2 h/day at age 2.5 years, and ST > 2 h/day at age 4 years; continuous low ST was defined as ST = 0 h/day at age 0.5 years, ST ≤ 2 h/day at age 2.5 years, and ST ≤ 2 h/day at age 4 years.

### Outcomes

The Strengths and Difficulties Questionnaire (SDQ) was completed by the parents and guardians to report on their children’s emotional and behavioral problems at age 4 years of age. The SDQ comprises 25 items. The possible scores of each item are scored as 0 (“not true”), 1 (“partialy true”), and 2 points (“completely true”), respectively. The questionnaire is divided into five dimensions: emotional symptoms, conduct problems, hyperactivity/attention deficit, peer problems, and prosocial behavior. The total difficulties’ score ranging from 0 to 40 is obtained using the sum of the scores on emotional symptoms, conduct problems, hyperactivity/attention deficit, and peer problems, with a higher score indicating a represents the higher the degree of objective difficulty. According to the cutoff scores adopted by Goodman et al. [20], children with total difficulties scores between 0 and 13 are defined as normal, 14 and 16 borderline, and 17 and 40 abnormal. For the subscales, the following cut-off values were applied: emotional problems: 0–3 = normal, 4 = borderline, 5–10 = abnormal; conduct problems: 0–2 = normal, 3 = borderline, 4–10 = abnormal; hyperactivity/inattention: 0–5 = normal, 6 = borderline, 7–10 = abnormal; peer problems: 0–2 = normal, 3 = borderline, 4–10 = abnormal; and prosocial behavior: 6–10 = normal, 5 = borderline, 0–4 = abnormal. We divided the scores of each sub-scale into normal (including normal and borderline categories) and abnormal groups. The internal consistency of the total difficulties score was good (Cronbach’s *α* = 0.784), as demonstrated in a previous study in China [21].

### Covariates

The socio-demographic variables of our study were as follows: age, gender (male or female), presence of siblings (yes or no), maximum educational level of parents (which was classified as the highest educational level of the parent who had more formal education; high school or less, college or 2-year degree, and bachelor’s or above), family income (< 2500 CNY/month, 2500–4000 CNY/month, > 4000 CNY/month), passive smoking (yes or no), outdoor activities (< 2 h/day, ≥ 2 h/day). In addition, information on the birth of the children was also reported. These variables included gestational weeks (full term or premature), delivery mode (vaginal delivery or cesarean section), and birth weight (small for gestational age, appropriate for gestational age, and large for gestational age).

### Statistical analysis

Chi-square tests were performed to test for the prevalence of prevalence of emotional and behavioral problems. Multiple logistic regression models were used to explore the independent associations between the ST at each age group and the SDQ scores. In additional analyses, the study examined the associations between sustained ST and emotional and behavioral problems which were also analyzed, which were dichotomized into normal (including normal and borderline categories) and abnormal groups. Meanwhile, the analysis was controlled for some potential confounders including gender, age, number of siblings, maximum educational level of parents, family income, passive smoking, outdoor activities, delivery mode, and birth weight. Data analysis were analyzed using the software SPSS, version 23.0. *P* < 0.05 was considered statistically significant.

## Results

### Characteristics of the sample

The distribution of the SDQ total and subscale in the sample are presented in Table 1. The abnormal proportion of total difficulties, emotional symptoms, conduct problems, hyperactivity, peer problems, and prosocial behavior were 10.8, 8.4, 9.8, 16.2, 26.1, and 12.1%, respectively. The abnormal proportion of emotional symptoms in girls was higher than in boys, while the abnormal proportion of total difficulties, conduct problems, hyperactivity problems, peer problems, and prosocial behavior in boys were higher than in girls. Children with low parental education levels and exposure to passive smoking were more likely to report emotional and behavioral problems.

**Table 1:** Distribution of emotional and behavioral problems (*n* = 2490)

Demographic factors	*n* (%)	Total difficulties		Emotional symptoms		Conduct problems		Hyperactivity		Peer problems		Prosocial behavior	
		*n* (% )	*χ*^2^ value	*n* (% )	*χ*^2^ value	*n* (% )	*χ*^2^ value	*n* (% )	*χ*^2^ value	*n* (% )	*χ*^2^ value	*n* (% )	*χ*^2^ value
Age (years)			0.41		2.47		0.25		0.24		1.71		0.36
<4	538 (21.6)	62 (11.5)		36 (6.7)		56 (10.4)		91 (16.9)		152 (28.3)		61 (11.3)	
≥4	1952 (78.4)	206 (10.6)		172 (8.0)		189 (9.7)		313 (16.0)		497 (25.5)		240 (12.3)	
Gender			7.57**		3.92*		4.74*		16.07**		14.74**		18.73**
Male	1289 (51.8)	160 (12.4)		94 (7.3)		143 (11.1)		246 (19.1)		378 (29.3)		191 (14.8)	
Female	1201 (48.2)	108 (9.0)		114 (9.5)		102 (8.5)		158 (13.2)		271 (22.6)		110 (9.2)	
Any siblings			0.38		0.65		0.30		0.21		0.18		0.79
Yes	269 (10.8)	26 (9.7)		19 (7.1)		29 (10.8)		41 (15.2)		73 (27.1)		37 (13.8)	
No	2221 (89.2)	242 (10.9)		189 (8.5)		216 (9.7)		363 (16.3)		576 (25.9)		264 (11.9)	
Delivery mode			0.37		1.32		0.05		2.01		0.02		0.37
Vaginal delivery	1233 (49.5)	128 (10.4)		95 (7.7)		122 (9.7)		187 (15.2)		320 (26.0)		152 (12.5)	
Cesarean section	1257 (50.4)	140 (11.1)		113 (9.0)		123 (10.0)		217 (17.3)		329 (26.2)		147 (11.7)	
Birth weight			0.95		0.05		0.14		1.76		0.31		0.43
Small for gestational age	247 (9.9)	25 (10.1)		21 (8.5)		23 (9.3)		44 (17.8)		68 (27.5)		30 (12.1)	
Appropriate for gestational age	1847 (74.1)	195 (10.6)		155 (8.4)		184 (10.0)		289 (15.6)		478 (25.9)		227 (12.3)	
Large for gestational age	396 (15.9)	48 (12.1)		32 (8.1)		38 (9.6)		71 (17.9)		103 (26.0)		44 (11.1)	
Family income (CNY/month)			2.15		1.93		4.87		0.33		9.46**		8.20*
< 2500	698 (28.0)	79 (11.3)		64 (9.2)		79 (11.3)		118 (16.9)		212 (30.4)		105 (15.0)	
2500–4000	1045 (42.0)	119 (11.4)		90 (8.6)		87 (8.3)		167 (16.0)		258 (24.7)		111 (10.6)	
≥ 4000	747 (30.0)	70 (9.4)		54 (7.2)		79 (10.6)		119 (15.9)		179 (24.0)		85 (11.4)	
Maximum educational level of parents			14.36**		5.56		14.07**		31.70***		19.68**		5.49
High school or less	758 (30.4)	107 (14.1)		77 (10.2)		100 (13.2)		169 (22.3)		241 (31.8)		109 (14.4)	
College or 2-year degree	787 (31.6)	65 (8.3)		54 (6.9)		69 (8.8)		118 (15.0)		195 (24.8)		85 (10.8)	
Bachelor’s or above	945 (38.0)	96 (10.2)		77 (8.1)		76 (8.0)		117 (12.4)		213 (22.5)		107 (11.3)	
Passive smoking			5.49*		9.82**		5.65*		8.46**		1.13		1.01
Yes	397 (15.9)	56 (14.1)		49 (12.3)		52 (13.1)		84 (21.2)		112 (28.2)		42 (10.6)	
No	2093 (84.1)	212 (10.1)		159 (7.6)		193 (9.2)		320 (15.3)		537 (25.7)		259 (12.4)	
Outdoor activities (h/day)			0.17		0.98		0.02		0.79		7.98**		3.52
< 2	1355 (54.4)	149 (11.0)		120 (8.9)		133 (9.8)		228 (16.8)		384 (28.3)		179 (13.2)	
≥ 2	1135 (45.6)	119 (10.5)		88 (7.8)		112 (9.9)		176 (15.5)		265 (23.3)		122 (10.7)	

*P < 0.05; **P < 0.01

### Associations between ST and follow-up emotional and behavioral problems

Table 2 reports the odds ratio of the relationship between ST and emotional and behavioral problems. The percentages of children with ST > 0 h/day at age 0.5 years, ST > 2 h/day at age 2.5 years, and ST > 2 h/day at age 4 years were 45.7, 55.5, and 34.5% respectively. The normal group was used as the control group. After adjusting for confounding factors, the high level of ST (> 0 h/day) at age 0.5 years was found to be a risk factor for abnormal emotional symptoms and hyperactivity, the high level of ST (> 2 h/day) at age 2.5 years a risk factor for abnormal hyperactivity, and the high level of ST (> 2 h/day) at age 4 years a risk factor for abnormal total difficulties, conduct problems, peer problems, hyperactivity, and prosocial behavior.

**Table 2:** Odds ratios (95% confidence intervals) of emotional and behavioral problems associated with screen time (*n* = 2490)

SDQ	ST at age of 0.5		ST at age of 2.5		ST at age of 4	
	0 h/day	> 0 h/day	≤ 2 h/day	> 2 h/day	≤ 2 h/day	> 2 h/day
No. of participants (%)	1353 (54.3)	1137 (45.7)	1108 (44.5)	1382 (55.5)	1630 (65.5)	860 (34.5)
Total difficulties						
Crude model	Ref	1.30 (1.01–1.67)*	Ref	1.34 (1.04–1.74)*	Ref	1.76 (1.36–2.28)**
Adjusted model^a^	Ref	1.23 (0.95–1.60)^b^	Ref	1.29 (0.98–1.68)	Ref	1.72 (1.31–2.24)**
Emotional symptoms						
Crude model	Ref	1.43 (1.07–1.90)*	Ref	1.10 (0.82–1.46)	Ref	1.43 (1.07–1.91)*
Adjusted model	Ref	1.36 (1.02–1.83)*	Ref	1.06 (0.79–1.43)	Ref	1.38 (0.99–1.90)
Conduct problems						
Crude model	Ref	1.41 (1.08–1.84)*	Ref	1.29 (0.99–1.70)	Ref	1.95 (1.49–2.54)**
Adjusted model	Ref	1.29 (0.99–1.70)	Ref	1.20 (0.91–1.58)	Ref	1.81 (1.38–2.39)**
Hyperactivity						
Crude model	Ref	1.43 (1.16–1.77)**	Ref	1.42 (1.14–1.76)**	Ref	1.52 (1.22–1.89)**
Adjusted model	Ref	1.31 (1.06–1.64)*	Ref	1.31 (1.04–1.63)*	Ref	1.38 (1.10–1.74)**
Peer problems						
Crude model	Ref	1.21 (0.93–1.34)	Ref	1.12 (0.94–1.34)	Ref	1.39 (1.16–1.67)**
Adjusted model	Ref	1.04 (0.87–1.25)	Ref	1.05 (0.87–1.27)	Ref	1.36 (1.12–1.65)**
Prosocial behavior						
Crude model	Ref	1.00 (0.79–1.28)	Ref	0.99 (0.78–1.27)	Ref	1.43 (1.11–1.82) **
Adjusted model	Ref	0.97 (0.75–1.24)	Ref	0.96 (0.75–1.23)	Ref	1.46 (1.13–1.89) **

*SDQ* Strength and Difficulties Questionnaire

^a^Adjustment for age, gender, number of siblings, delivery model, birth weight, maximum educational level of parents, family income, passive smoking, outdoor activities

^b^*OR* odd ratio, *CI* confidence interval

*P < 0.05; ** *P* < 0.01

### The gender difference between ST and emotional and behavioral problems

The gender differences between ST and the abnormal of emotional and behavioral problems are shown in Table 3. There were a positive associations between ST at age 0.5 years and emotional symptoms (OR = 1.55, 95% CI 1.04–2.32), conduct problems (OR = 1.54, 95% CI 1.01–2.35) in girls, and hyperactivity (OR = 1.36, 95% CI 1.02–1.80) in boys. The associations between ST at age 4 years and total difficulties (OR = 1.62, 95% CI 1.14–2.30), conduct problems (OR = 1.76, 95% CI 1.22–2.54), hyperactivity (OR = 1.41, 95% CI 1.05–1.89), peer problems (OR = 1.43, 95% CI 1.11–1.85), and prosocial behavior (OR = 1.62, 95% CI 1.17–2.24) were found in boys, and total difficulties (OR = 1.89, 95% CI 1.25–2.87) and conduct problems (OR = 1.96, 95% CI 1.28–3.00) were found in girls. There were no correlation between the ST at age 2.5 years and emotional and behavioral problems in both boys and girls. No gender differences were found in the independent effects of ST on emotional and behavioral problems.

**Table 3:** Odds ratios (95% confidence intervals) of emotional and behavioral problems associated with screen time stratified by gender (*n* = 2490)

SDQ	ST at age of 0.5		ST at age of 2.5		ST at age of 4	
	0 h/day	> 0 h/day	≤ 2 h/day	> 2 h/day	≤ 2 h/day	> 2 h/day
Total difficulties						
Boys ^a^	Ref	1.12 (0.79–1.57)^b^	Ref	1.23 (0.87–1.75)	Ref	1.62 (1.14–2.30)**
Girls ^a^	Ref	1.38 (0.92–2.09)	Ref	1.39 (0.91–2.11)	Ref	1.89 (1.25–2.87)**
ROR		0.81 (0.48–1.39)		0.88 (0.51–1.53)		0.86 (0.50–1.48)
Emotional symptoms						
Boys	Ref	1.15 (0.75–1.78)	Ref	1.09 (0.73–1.63)	Ref	1.39 (0.89–2.16)
Girls	Ref	1.55 (1.04–2.32)*	Ref	1.04 (0.69–1.55)	Ref	1.47 (0.97–2.22)
ROR		0.74 (0.41–1.34)		1.05 (0.59–1.85)		0.95 (0.52–1.73)
Conduct problems						
Boys	Ref	1.12 (0.78–1.60)	Ref	1.17 (0.82–1.69)	Ref	1.76 (1.22–2.54)**
Girls	Ref	1.54 (1.01–2.35)*	Ref	1.26 (0.83–1.94)	Ref	1.96 (1.28–3.00)**
ROR		0.73 (0.42–1.27)		0.93 (0.53–1.62)		0.90 (0.51–1.58)
Hyperactivity						
Boys	Ref	1.36 (1.02–1.80)*	Ref	1.27 (0.95–1.71)	Ref	1.41 (1.05–1.89)*
Girls	Ref	1.26 (0.89–1.78)	Ref	1.36 (0.96–1.95)	Ref	1.35 (0.95–1.94)
ROR		1.08 (0.69–1.69)		0.93 (0.59–1.48)		1.04 (0.66–1.66)
Peer problems						
Boys	Ref	1.12 (0.88–1.44)	Ref	0.95 (0.74–1.23)	Ref	1.43 (1.11–1.85)**
Girls	Ref	0.94 (0.71–1.24)	Ref	1.21 (0.91–1.60)	Ref	1.28 (0.96–1.73)
ROR		1.19 (0.82–1.73)		0.79 (0.54–1.15)		1.12 (0.76–1.65)
Prosocial behavior						
Boys	Ref	0.83 (0.61–1.14)	Ref	1.01 (0.73–1.38)	Ref	1.62 (1.17–2.24)**
Girls	Ref	1.23 (0.82–1.84)	Ref	0.90 (0.60–1.35)	Ref	1.26 (0.83–1.93)
ROR		0.67 (0.40–1.12)		1.12 (0.67–1.88)		1.29 (0.75–2.19)

*SDQ* Strength and Difficulties Questionnaire, *ROR* ratio of two odds ratio in boys versus girls

^a^Adjustment for age, number of siblings, delivery model, birth weight, maximum educational level of parents, family income, passive smoking, outdoor activities

^b^*OR* odd ratio, *CI* confidence interval

*P < 0.05; **P < 0.01

### Associations between sustained ST and emotional and behavioral problems

**Fig. 2:**
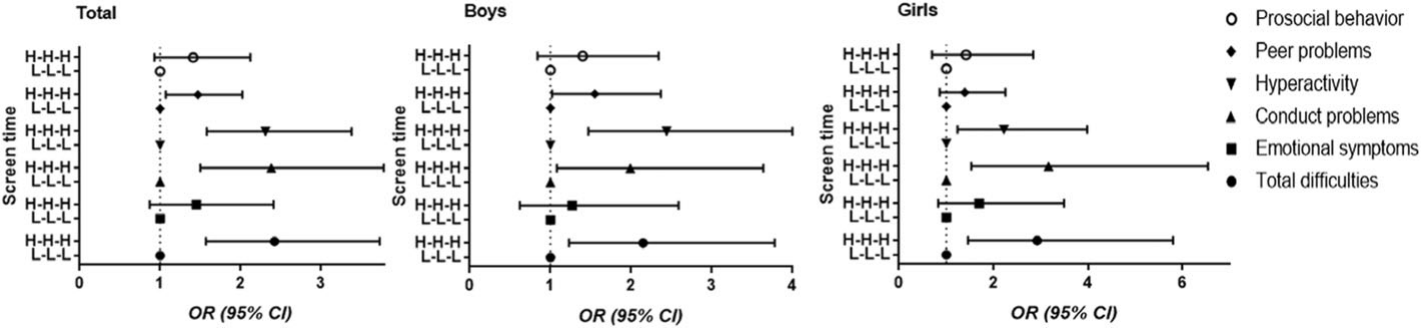
Adjusted odds ratios (95% confidence intervals) between sustained ST and emotional and behavioral problems (H-H-H, continuous high ST; L-L-L, continuous low ST)

Children with continuous high ST had a significantly increased risk for the abnormal of total difficulties, conduct problems, hyperactivity, and peer problems compared to children with continuous low ST, and no gender differences were found, either (Fig. 2).

## Discussion

This study demonstrates that high ST at 0.5, 2.5, and 4 years of age may be important predictors of the emotional and behavioral problems at 4 years of age. In addition, sustained high ST is more likely to lead to behavioral problems when compared to with sustained low ST after controlling for potential confounders.

In our study, almost half of the participants were exposed to electronic screens at 6 months of age. The percentages of children who did not follow the recommended of AAP regarding ST at ages 2.5 and 4 years were 55.5 and 34.5%, respectively. The decrease in percentages with increasing age may be due to children entrance kindergarten. Similarly, a British study showed that nearly two thirds of 5-year-old children watch television for 1 to 3 h/day, while 15% of them watch television for more than 3 h/day [9]. The effects of ST on children are extensive, children are still immature and more likely to have emotional and behavioral problems affected by electronic media. Meanwhile, children’s emotional and behavioral problems were high (10.8%) in this Chinese birth cohort, which is inferior to Australia (14%) [22] and South Korea (11.8%) [23] and similar to Japan (11%) [24]. The prevalence of emotional and behavioral problems among preschool children in our study was also higher than in other provinces (9.6%) of China [25]. These differences in findings may be due to the diversification of social and cultural backgrounds in different countries and regions and the different classification criteria of questionnaires. Children with low parental educational levels and those who are exposed to passive smoking are more likely to report emotional and behavioral problems. Therefore, attention should be paid regarding the impact of the family environment and exposure to secondhand smoking on children’s health [26].

Previous studies have shown that high levels of ST are associated with poorer outcomes, such as aggression [27], inattention problems [28], prosocial behavior [29], and emotional problems [30], which are consistent with our findings. We found negative effects of high ST at an earlier age on later emotional and behavioral problems. Few longitudinal studies have investigated the association between ST for infants and toddlers and the risk of emotional and behavioral problems. A prospective cohort study showed that the longer children use electronic media in early childhood, the worse their well-being, but this was concluded using the emotional symptoms and peer problems subscale of the SDQ [31]. We found that the exposure to electronic media has a negative effect on children’s attention problems at each age group. Previous studies have shown that exposure to television screens in children aged 18 months can impair attention at age 30 months [29], and exposure to higher levels of electronic media at age 29 months can predict poor academic outcomes for school-age children, which requires concentration in class [32]. Furthermore, early childhood video behavior is considered to have developmental continuity [33], and 16.5% of children in our study sustained a high screen time. We also found that the negative effect of sustained high ST exposure from 6 months to 4 years of age is stronger than that of concurrent exposure or early exposure only, consistent with the results of Mistry et al.’s study [34], and sustained exposure is a risk factor for behavioral problems. Therefore, the findings of this study reinforce the negative impact of electronic media use on children’s psychological health.

Gender differences were identified in the occurrence of emotional and behavioral problems. In line with prior studies, boys were more prone to report total difficulties, conduct problems, hyperactivity problems, peer problems, and prosocial behavior than girls, while girls were more likely than boys to report emotional problems [35, 36]. In addition, there were no gender differences in the health effects of early childhood electronic media exposure in subsequent emotional and behavioral problems, which is inconsistent with the findings of Hinkley et al.’s study [31]. But we can see that girls seem to be more susceptible to screen time than boys. The mechanism of these gender differences has not been well understood and may be due to different levels of trait neuroticism; girls tend to ruminate more frequently than boys [37].

The mechanism of ST on emotional and behavioral problems is unclear. Some studies suggest that, according to the social learning theory, children can easily learn what is on a screen by observing and imitating [38]. In addition, the stimulation of electronic media for children is so diverse, and the focus of most programs changes rapidly, which can impair children’s ability to concentrate [16]. An animal model study suggested that excessive visual and auditory stimulation of newborn mice can have a significant adverse effect on its subsequent neurocognitive function [39]. Nonetheless, additional research is needed to further investigate such potential mechanisms.

The strengths of the study is its being a longitudinal study and that it analyzed boys and girls separately. The survey was conducted by maternal and child healthcare professionals, and the questionnaire was authentic and complete. Except for general demographic sociological characteristics, pregnancy and birth data were also included as potential confounders. However, there are still some limitations to this study. First, ST were reported by parents and thus the data may have been inaccurate, despite this, however, as most of the ST exposure of infants and young children occurs at home, there is good reason to believe that ST was accurately measured. Second, there are many factors that may affect children’s emotional and behavioral problems which were not included in this study as controlled factors, such as maternal emotional problems [40] and parenting style [41], we have not controlled these factors. Third, the occurrence of selection bias due to loss-to-follow-up also limits our study. At 4 years of age, 2492 children were included in the study, the follow-up rate was 76.2%. Baseline birth information and general demographic characteristics of the lost and followed children were analyzed, and the results showed that there were no significant differences in gender and parental education. In addition, we did not consider the impact of video content, and previous studies have shown that electronic media content were associated with children’s attention problems [42] and aggressive behavior [27].

## Conclusions

This study indicates that children with high ST exposure at an early age are prone to emotional and behavioral problems. In particular, sustained high ST exposure is a risk factor for behavioral problems. Therefore, parents and kindergarten should pay attention to limiting the ST of children. Further research is also needed to explore the possible mechanisms of this association.

## Supplementary Information

**Additional file 1: Table S1.** Demographic characteristics between loss-to-follow up and follow up groups. **Table S2.** Odds ratios (95% confidence intervals) of emotional and behavioral problems associated with screen time (normal only vs. abnormal groups). **Table S3.** Odds ratios (95% confidence intervals) of emotional and behavioral problems associated with screen time stratified by gender (normal only vs. abnormal groups). **Table S4.** Adjusted odds ratios (95% confidence intervals) between sustained ST and emotional and behavioral problems (normal only vs. abnormal groups).

## Data Availability

The data used to derive our conclusions are unsuitable for public deposition due to ethical restrictions imposed by the institutional ethics committee, as the data contain sensitive information on participants and facilities.
