# Supplementary material for: Early childhood screen time as a predictor of emotional and behavioral problems in children at 4 years: a birth cohort study in China
Source: Environ Health Prev Med. 2021 Jan 7;26:3. doi: 10.1186/s12199-020-00926-w (PMC7789634; doi:10.1186/s12199-020-00926-w)
Supplement: Supplementary file 1 — Additional file 1: Table S1. Demographic characteristics between loss-to-follow up and follow up groups. Table S2. Odds ratios (95% confidence intervals) of emotional and behavioral problems associated with screen time (normal only vs. abnormal groups). Table S3. Odds ratios (95% confidence intervals) of emotional and behavioral problems associated with screen time stratified by gender (normal only vs. abnormal groups). Table S4. Adjusted odds ratios (95% confidence intervals) between sustained ST and emotional and behavioral problems (normal only vs. abnormal groups). [file 12199_2020_926_MOESM1_ESM.doc]

**Table S1** Demographic characteristics between loss-to-follow up and follow up groups.

| Demographic factors | n (%) | loss-to-follow up | follow up | $\chi^{2}$value | *P* value |
| --- | --- | --- | --- | --- | --- |
| Gender |  |  |  | 1.64 | 0.200 |
| Male | 1668(51.0) | 379(48.7) | 1289(51.7) |  |  |
| Female | 1602(49.0) | 399(51.2) | 1203(48.3) |  |  |
| Delivery mode |  |  |  | 0.01 | 0.945 |
| Vaginal delivery | 1616(49.5) | 383(49.4) | 1233(49.5) |  |  |
| Caesarean section | 1650(50.5) | 393(50.6) | 1257(50.5) |  |  |
| Birth weight |  |  |  | 4.31 | 0.116 |
| small for gestational age | 316(9.7) | 69(8.9) | 247(9.9) |  |  |
| appropriate for gestational age | 2046(73.7) | 559(72.1) | 1847(74.2) |  |  |
| large for gestational age | 543(16.6) | 147(19.0) | 396(15.9) |  |  |
| Family income (CNY/month) |  |  |  | 12.55 | 0.002 |
| <2500 | 866(26.5) | 168(21.6) | 698(28.0) |  |  |
| 2500-4000 | 1400(42.8) | 354(45.5) | 1046(42.0) |  |  |
| ≥4000 | 1004(30.7) | 256(32.9) | 748(30.0) |  |  |
| Mother's education |  |  |  | 0.57 | 0.751 |
| Junior high school or less | 660(20.2) | 161(20.7) | 499(20.0) |  |  |
| High school | 735(22.5) | 180(23.1) | 555(22.3) |  |  |
| College or above | 1875(57.3) | 437(56.2) | 1438(57.7) |  |  |
| Father's education |  |  |  | 0.59 | 0.744 |
| Junior high school or less | 484(14.8) | 118(15.2) | 366(14.7) |  |  |
| High school | 910(27.8) | 223(28.7) | 687(27.6) |  |  |
| College or above | 1876(57.4) | 437(56.2) | 1439(57.7) |  |  |

**Table S2** Odds ratios (95% confidence intervals) of emotional and behavioral problems associated with screen time (normal only vs. abnormal groups)

| SDQ | ST at age of 0.5 | |  | ST at age of 2.5 | |  | ST at age of 4 | |
| --- | --- | --- | --- | --- | --- | --- | --- | --- |
|  | 0 h/day | > 0 h/day |  | ≤ 2 h/day | > 2 h/day |  | ≤ 2 h/day | ≥ 2 h/day |
| Total difficulties |  |  |  |  |  |  |  |  |
| Crude model | Ref | 1.36(1.05 - 1.75) * |  | Ref | 1.40(1.08 - 1.83) * |  | Ref | 1.92(1.48 - 2.49) ** |
| Adjusted model^a^ | Ref | 1.27(0.98 - 1.66) ^b^ |  | Ref | 1.32(1.02 - 1.73)* |  | Ref | 1.85(1.41 - 2.42) ** |
| Emotional symptoms |  |  |  |  |  |  |  |  |
| Crude model | Ref | 1.45(1.09 - 1.93) * |  | Ref | 1.11(0.83 - 1.48) |  | Ref | 1.47(1.10 - 1.97) ** |
| Adjusted model | Ref | 1.36(1.02 - 1.83) * |  | Ref | 1.07(0.80 - 1.44) |  | Ref | 1.45(1.07 - 1.96)* |
| Conduct problems |  |  |  |  |  |  |  |  |
| Crude model | Ref | 1.46(1.12 - 1.91) ** |  | Ref | 1.32(1.01 - 1.74)* |  | Ref | 2.01(1.53 - 2.63) ** |
| Adjusted model | Ref | 1.34(1.02 - 1.76) * |  | Ref | 1.21(0.92 - 1.60) |  | Ref | 1.85(1.39 - 2.44) ** |
| Hyperactivity |  |  |  |  |  |  |  |  |
| Crude model | Ref | 1.46(1.17 - 1.81) ** |  | Ref | 1.44(1.15 - 1.80) ** |  | Ref | 1.60(1.28 - 2.00) ** |
| Adjusted model | Ref | 1.33(1.06 - 1.66) * |  | Ref | 1.32(1.05 - 1.65) * |  | Ref | 1.46(1.15 - 1.84)** |
| Peer problems |  |  |  |  |  |  |  |  |
| Crude model | Ref | 1.16(0.96 - 1.41) |  | Ref | 1.14(0.95 - 1.38) |  | Ref | 1.42(1.17 - 1.73) ** |
| Adjusted model | Ref | 1.07(0.88 - 1.30) |  | Ref | 1.05(0.86 - 1.28) |  | Ref | 1.36(1.10 - 1.67) ** |
| Prosocial behavior |  |  |  |  |  |  |  |  |
| Crude model | Ref | 0.98(0.77 - 1.26) |  | Ref | 1.00(0.78 - 1.27) |  | Ref | 1.43(1.11 - 1.83) ** |
| Adjusted model | Ref | 0.96(0.75 - 1.24) |  | Ref | 0.96(0.74 - 1.13) |  | Ref | 1.51(1.16 - 1.96) ** |

Strength and Difficulties Questionnaire (SDQ).

^a^ Adjustment for age, gender, number of siblings, delivery model, birth weight, maximum educational level of parents, family income, passive smoking ,[o](../../../../D:/%25E6%259C%2589%25E9%2581%2593%25E8%25AF%258D%25E5%2585%25B8/Dict/8.9.3.0/resultui/html/index.html#/javascript:;)utdoor [activities](../../../../D:/%25E6%259C%2589%25E9%2581%2593%25E8%25AF%258D%25E5%2585%25B8/Dict/8.9.3.0/resultui/html/index.html#/javascript:;). ^b^ *OR* odd ratio, *CI* confidence interval.

*P < 0.05; ** P < 0.01.

**Table** **S3** Odds ratios (95% confidence intervals) of emotional and behavioral problems associated with screen time stratified by gender(normal only vs. abnormal groups)

| SDQ | ST at age of 0.5 | | |  | ST at age of 2.5 | |  | ST at age of 4 | | |
| --- | --- | --- | --- | --- | --- | --- | --- | --- | --- | --- |
|  | 0 h/day | | > 0 h/day |  | ≤ 2 h/day | > 2 h/day |  | ≤ 2 h/day | | ≥ 2 h/day |
| Total difficulties |  |  | |  |  |  |  |  |  | |
| Boys ^a^ | Ref | 1.17(0.83 - 1.66)^b^ | |  | Ref | 1.26(0.88 - 1.79) |  | Ref | 1.81(1.27 - 2.58)** | |
| Girls ^a^ | Ref | 1.41(0.93 - 2.14) | |  | Ref | 1.44(0.95 - 2.20) |  | Ref | 1.94(1.28 - 2.96)** | |
| Emotional symptoms |  |  | |  |  |  |  |  |  | |
| Boys | Ref | 1.18(0.76 - 1.81) | |  | Ref | 1.05(0.68- 1.63) |  | Ref | 1.45(0.92 - 2.27) | |
| Girls | Ref | 1.52(1.03 - 2.29)* | |  | Ref | 1.10(0.74 - 1.65) |  | Ref | 1.50(0.99 - 2.27) | |
| Conduct problems |  |  | |  |  |  |  |  |  | |
| Boys | Ref | 1.20(0.83 - 1.72) | |  | Ref | 1.19(0.82 - 1.74) |  | Ref | 1.82(1.25- 2.65)** | |
| Girls | Ref | 1.53(0.99 - 2.34) | |  | Ref | 1.27(0.83 - 1.95) |  | Ref | 1.92(1.25 - 2.96)** | |
| Hyperactivity |  |  | |  |  |  |  |  |  | |
| Boys | Ref | 1.40(1.05 - 1.88)* | |  | Ref | 1.28(0.95 - 1.73) |  | Ref | 1.50(1.11 - 2.04)** | |
| Girls | Ref | 1.23(0.86 - 1.74) | |  | Ref | 1.39(0.97 - 1.99) |  | Ref | 1.41(0.99 - 2.02) | |
| Peer problems |  |  | |  |  |  |  |  |  | |
| Boys | Ref | 1.18(0.90 - 1.54) | |  | Ref | 0.94(0.72 - 1.24) |  | Ref | 1.46(1.11 - 1.93)** | |
| Girls | Ref | 0.94(0.70 - 1.26) | |  | Ref | 1.20(0.89 - 1.61) |  | Ref | 1.26(0.93 - 1.73) | |
| Prosocial behavior |  |  | |  |  |  |  |  |  | |
| Boys | Ref | 0.82(0.59 - 1.14) | |  | Ref | 0.99(0.72 - 1.37) |  | Ref | 1.69(1.21 - 2.37)** | |
| Girls | Ref | 1.22(0.81 - 1.83) | |  | Ref | 0.93(0.62 - 1.39) |  | Ref | 1.26(0.82 - 1.93) | |

Strength and Difficulties Questionnaire (SDQ).

^a^ Adjustment for age, number of siblings, delivery model, birth weight, maximum educational level of parents, family income, passive smoking ,[o](../../../../D:/%25E6%259C%2589%25E9%2581%2593%25E8%25AF%258D%25E5%2585%25B8/Dict/8.9.3.0/resultui/html/index.html#/javascript:;)utdoor [activities](../../../../D:/%25E6%259C%2589%25E9%2581%2593%25E8%25AF%258D%25E5%2585%25B8/Dict/8.9.3.0/resultui/html/index.html#/javascript:;). ^b^ *OR* odd ratio, *CI* confidence interval.

*P < 0.05; ** P < 0.01.

**Table** **S4** Adjusted odds ratios (95% confidence intervals) between sustained ST and emotional and behavioral problems(normal only vs. abnormal groups).

| Sustained ST | SDQ | | | | | |
| --- | --- | --- | --- | --- | --- | --- |
|  | Total difficulties | Emotional symptoms | Conduct problems | Hyperactivity | Peer problems | Prosocial behavior |
| All |  |  |  |  |  |  |
| L-L-L^a^ | Ref | Ref | Ref | Ref | Ref | Ref |
| H-H-H | 2.70(1.75 - 4.17)** | 1.64(0.99 - 2.70)^b^ | 2.45(1.54 - 3.90)** | 2.47(1.68 - 3.63)** | 1.42(1.01 - 0.97)* | 1.27(0.84 - 1.94) |
| Boys |  |  |  |  |  |  |
| L-L-L | Ref | Ref | Ref | Ref | Ref | Ref |
| H-H-H | 2.39(1.35 - 4.22)** | 1.49(0.74 - 2.99) | 2.10(1.14 - 3.90)* | 2.55(1.52 - 4.26)** | 1.53(0.98 - 2.39) | 1.25(0.74 - 2.13) |
| Girls |  |  |  |  |  |  |
| L-L-L | Ref | Ref | Ref | Ref | Ref | Ref |
| H-H-H | 3.28(1.66 - 6.48)** | 1.84(0.90 - 3.73) | 3.10(1.51 - 6.41)* | 2.42(1.35 - 4.32)** | 1.33(0.80 - 2.21) | 1.33(0.66 - 2.67) |

Strength and Difficulties Questionnaire (SDQ).

H-H-H: continuous high ST; L-L-L: continuous low ST.

^a^ Adjustment for age, gender, number of siblings, delivery model, birth weight, maximum educational level of parents, family income, passive smoking ,[o](../../../../D:/%25E6%259C%2589%25E9%2581%2593%25E8%25AF%258D%25E5%2585%25B8/Dict/8.9.3.0/resultui/html/index.html#/javascript:;)utdoor [activities](../../../../D:/%25E6%259C%2589%25E9%2581%2593%25E8%25AF%258D%25E5%2585%25B8/Dict/8.9.3.0/resultui/html/index.html#/javascript:;). ^b^ *OR* odd ratio, *CI* confidence interval.

*P < 0.05; ** P < 0.01.
